# Modeling the quantitative nature of neurodevelopmental disorders using Collaborative Cross mice

**DOI:** 10.1186/s13229-018-0252-2

**Published:** 2018-12-13

**Authors:** Remco T. Molenhuis, Hilgo Bruining, Myrna J. V. Brandt, Petra E. van Soldt, Hanifa J. Abu-Toamih Atamni, J. Peter H. Burbach, Fuad A. Iraqi, Richard F. Mott, Martien J. H. Kas

**Affiliations:** 1 Department of Translational Neuroscience, Brain Center Rudolf Magnus, University Medical Center Utrecht, Universiteitsweg 100, 3584 CG Utrecht, The Netherlands; 2 Department of Psychiatry, Brain Center Rudolf Magnus, University Medical Center Utrecht, Heidelberglaan 100, 3584 CX Utrecht, The Netherlands; 3 Department of Clinical Microbiology and Immunology, Sackler Faculty of Medicine, Tel Aviv University, Ramat Aviv, 69978 Tel Aviv, Israel; 4 Genetics Institute, University College London, Gower Street, London, WC1E 6BT UK; 5 Groningen Institute for Evolutionary Life Sciences, University of Groningen, Nijenborgh 7, 9747 AG Groningen, The Netherlands

**Keywords:** Neurodevelopmental disorders, Autism, Animal models, Quantitative genetics, Genetic reference population, Behavioral neuroscience, Histamine 3 receptor, Repetitive behavior

## Abstract

**Background:**

Animal models for neurodevelopmental disorders (NDD) generally rely on a single genetic mutation on a fixed genetic background. Recent human genetic studies however indicate that a clinical diagnosis with ASDAutism Spectrum Disorder (ASD) is almost always associated with multiple genetic fore- and background changes. The translational value of animal model studies would be greatly enhanced if genetic insults could be studied in a more quantitative framework across genetic backgrounds.

**Methods:**

We used the Collaborative Cross (CC), a novel mouse genetic reference population, to investigate the quantitative genetic architecture of mouse behavioral phenotypes commonly used in animal models for NDD.

**Results:**

Classical tests of social recognition and grooming phenotypes appeared insufficient for quantitative studies due to genetic dilution and limited heritability. In contrast, digging, locomotor activity, and stereotyped exploratory patterns were characterized by continuous distribution across our CC sample and also mapped to quantitative trait loci containing genes associated with corresponding phenotypes in human populations.

**Conclusions:**

These findings show that the CC can move animal model studies beyond comparative single gene-single background designs, and point out which type of behavioral phenotypes are most suitable to quantify the effect of developmental etiologies across multiple genetic backgrounds.

**Electronic supplementary material:**

The online version of this article (10.1186/s13229-018-0252-2) contains supplementary material, which is available to authorized users.

## Background

Recent studies show that genetic risk factors for neurodevelopmental disorders (NDD) such as autism spectrum disorder (ASD) and attention deficit hyperactivity disorder (ADHD) segregate in the general population. They influence continua of behavioral and developmental traits [1–4]. These findings confirm the clinical notion that the extreme tails of trait distributions are most likely to be associated with a clinical diagnosis and that genetic risk factors for ASD and ADHD confer quantitative rather than categorical effects [5–7]. The important implication of these studies is that continuous behavioral dimensions instead of dichotomous outcomes should be studied to acknowledge the quantitative effects of etiological factors.

This notion also has implications for animal studies into the biology of NDD. So far, animal models for NDD are generally designed to study the impact of a single genetic mutation in a fixed genetic background. Indeed, they have provided crucial insights into NDD biology and have transformed the landscape of treatment development [8]. However, recent human genetic studies indicate that ASD is most often not due to single genetic mutations, but rather occurs due to multiple genetic variants—even in individuals in which a contributing rare genetic mutation has been identified [4]. Another complexity is that genotype-phenotype relationships observed in a single mouse genetic background are often not generalizable to other backgrounds, thereby limiting their translational value [9]. To overcome these limitations, animal model strategies should also enable to evaluate the clinical impact of etiological factors on continuous behavioral traits across diverse genetic backgrounds.

Over the past century, a great deal of methods and resources has been optimized to study the genetic basis of quantitative physiological and neurobehavioral traits in animal model populations [10–12]. In a classical approach, genetic mapping of quantitative traits is carried out in an F2 intercross of two strains that are selected for divergent expression of a particular physiological or neurobehavioral trait [13, 14]. This approach was recently used in the context of autistic-like phenotypes, to identify quantitative trait loci (QTLs) underlying social interaction differences between C57BL/6J and the BTBR mouse model for autistic-like phenotypes [15] (also see [16–18]). Alternatively, outbred populations descendent from multiple inbred strains can be used to achieve high-resolution genetic mapping, by exploiting genetic recombination events that have accumulated over many generations of pseudo-random breeding [19]. Although this approach can be very effective to achieve high-resolution genetic mapping, a limitation is the need to genotype every individual animal, and the fact that data derived from a particular genetic background cannot be replicated. To study complex trait variation in more controlled genetic backgrounds, genetic reference populations (GRPs) are available, which are collections of inbred mouse strains each generated by repeated brother-sister mating for at least 20 generations. A particular type of GRPs constitute panels of recombinant inbred lines (RILs), such as mouse lines derived from C57BL/6J and DBA/2J founder strains, known as the BXD panel. BXD mouse lines have been widely used to study the genetic basis of complex neurobehavioral traits [20–22]; however, their genetic diversity is limited to the variation present in their two classical laboratory founder strains. Since classical laboratory strains were obtained after decades of human-driven artificial selection, inbreeding, and adaptation to captivity, RIL panels that include wild-derived genetic variation are expected to give unprecedented opportunities to study the genetic basis of fitness-driven behavioral responses [23, 24].

**Fig. 1:**
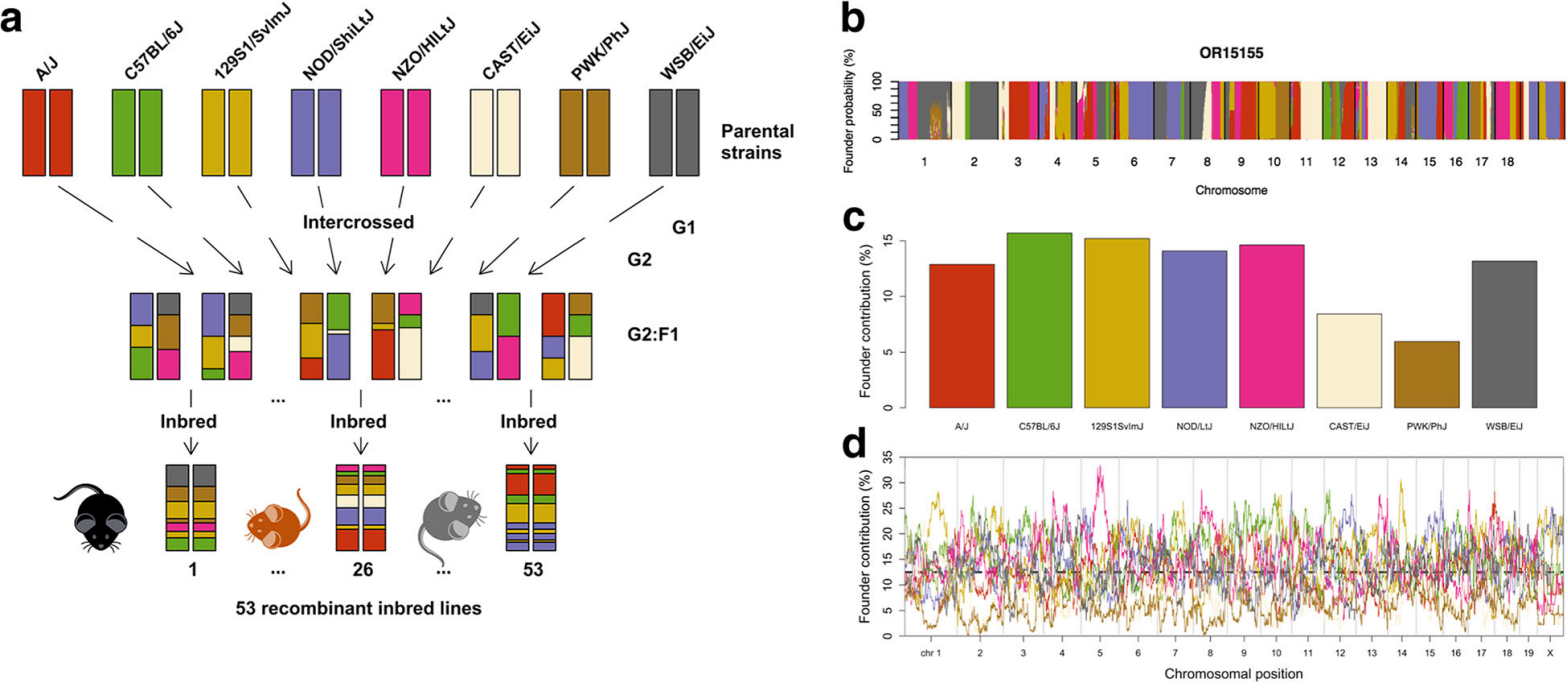
Genome structures across CC lines in this study. **a** CC lines used in this study were created from independent three-round intercrosses of eight parental founder strains, five of which are classical laboratory strains (C57BL/6J, A/J, 129S1/SvImJ, NOD/ShiLtJ, and NZO/HlLtJ) and three of which are wild-derived inbred strains (CAST/EiJ, PWK/PhJ, and WSB/EiJ). The resulting F1 mice were iteratively inbred and constitute a population of RI lines that are considered fully inbred at generation F20+. **b** Example of a genome reconstruction of a CC line, as a mosaic of the eight CC founder strains. Intervals of ambiguity are either caused by remaining heterozygosity or are regions where the founder strains have identical haplotypes. **c** Overall founder strain contributions in CC lines used for genetic analysis in this study, indicating lower abundance of CAST/EiJ, PWK/PhJ founder haplotypes. **d** Genome-wide founder strain contributions indicate absence of PWK/PhJ founder haplotypes at three genomic intervals, on chromosomes 2 and 8

We here tested the potential of the Collaborative Cross (CC), a mouse genetic reference population, and establish a quantitative framework to study the impact of etiological factors on neurobehavioral and ASD-like trait distributions. The CC is a panel of mouse recombinant inbred lines (RI lines) with a unique level of genetic diversity derived from five classical laboratory strains and three wild-derived mouse inbred lines (Fig. 1a) [25]. The resulting level of genetic diversity is considerably higher compared to existing RI panels, which is a crucial asset to match the human population studies into behavioral variation [25–28]. In this study, we used 53 CC lines with an average of 5–6 animals per lines. This design was based on previous studies in CC mice with similar or lower numbers that were found to have sufficient power to identify high-resolution QTLs underlying complex traits such as host susceptibility to infection, glucose tolerance, and polyp development [29–33]. Using 53 CC lines, we find that general physical and basic behavioral traits such as digging and locomotor activity show substantial continuous variation and heritability in CC lines. Stereotyped exploratory patterns were also characterized by strong genetic influences, in contrast to mouse phenotypes commonly studied to establish face validity for ASD. To confirm translational relevance of neurobehavioral and ASD-like trait variations in the CC population, we find that several of the loci mapped for neurobehavioral traits contained homologous genes implicated in ASD and brain developmental processes.

## Methods

### Collaborative Cross mice

We investigated neurobehavioral phenotypes in 53 Collaborative Cross RI lines available at Tel Aviv University (TAU), Israel. The CC lines were initially bred at three different locations from eight common founder strains [25]. These included 37 CC lines originating from the International Livestock Research Institute (ILRI) in Kenya and relocated to Tel Aviv University (Israel) in 2006, 10 CC lines originating from Geniad Ltd. in Western Australia (CC-GND), and six CC lines originating from the Oak Ridge National Laboratory (ORNL) in Tennessee that were subsequently relocated to the University of North Carolina in 2009 (CC-UNC). All 285 mice tested in this study were male and born, raised, and tested in the small animal facility at TAU. We focused on male mice because ASD is more common in male individuals than in females (by a ratio of 3:1) [34] and because the 4–5 day estrous cycle of female mice complicates reliable behavioral testing, in particular across different genetic backgrounds [35]. Animals were socially housed in type II Makrolon cages (26.5 × 17 × 11.4 cm) with a wood chip bedding, kept under 12-h light/dark cycle (6:00 am–6:00 pm) at 21–23 °C, and given tap water and rodent chow ad libitum.

### Reconstruction of the genomes of CC lines

Genome structures of CC lines were derived from SNPs array data from the Mouse Universal Genotyping Array (MUGA, 7500 markers) [27] and MegaMUGA (77,800 markers). For each CC line, we reconstructed the founder-based mosaic using the hidden Markov model (HMM) HAPPY package [31, 36] resulting in a probability matrix of descent from each CC founder that was subsequently pruned to about 11,000 SNPs.

### Social interaction and discrimination test

Social interaction and short- and long-term memory in a social context were measured during the social discrimination task, as performed previously [17, 37]. First, test animals were allowed to habituate to a clean and transparent mouse cage with wood chip bedding for 5 min. Then, they were exposed to an unfamiliar age- and gender-matched A/J conspecific for 2 min (T0). After an inter-trial interval (ITI) of 5 min, during which the mice stayed in the test cage, mice were exposed to the familiar conspecific and a first novel A/J conspecific for 2 min (short-term memory, T1). On day 2, after the 24-h ITI, the test animal was habituated for 5 min and re-exposed to the same familiar intruder of day 1 and to a different novel intruder animal from a different cage and housing room than the intruder of day 1 for 2 min (long-term memory, T24). The times spent exploring the stimulus animals was manually recorded using The Observer XT (Noldus Information Technology, Wageningen, The Netherlands). Social interaction was calculated as the total time exploring the unfamiliar conspecific during the 2-min interval T0. Discrimination capacity was calculated as the preference for the novel versus the familiar animal following the formula tN/(tN + tF), where tN is the time spent exploring the novel animal and tF is the time spent exploring the familiar animal. A ratio value above 0.5 indicated a successful discrimination between novel and familiar animal. Aggressive behaviors and or fighting resulted in experiments to be ceased as these behaviors interfere with reliable measurements of social interaction and discrimination capacity.

### General behavioral and morphological mouse phenotyping

On day 3 of the experiment, mouse behaviors including locomotor activity, exploratory patterns, digging, and grooming were assessed, while animals were exposed to four novel objects in a 26.5 × 17 × 11.4-cm arena with wood chip bedding. Digging and grooming behaviors were manually recorded using The Observer XT (Noldus Information Technology, Wageningen, The Netherlands). Locomotor trajectories were recorded using an overhead camera connected to a PC with video tracking software (EthoVision XT, Noldus Information Technology, Wageningen, The Netherlands) using LOWESS smoothing. These trajectories were then used to calculate the total distance moved and to investigate exploratory patterns. Stereotyped exploratory patterns were quantified as previously reported by Bonasera et al. [38] and as indicated in Additional file 1: Figure S3. Theme software version 5.0 (Noldus, Wageningen) was used to detect patterns and to calculate the total time in patterns. To correct for differences in locomotor activity, this analysis was repeated using equal locomotor distance per animal (i.e., repeating the analysis and averaging the results for the first and last 800 cm in CC mice). On day 4 of the experiment, animals were euthanized, and brain tissues were isolated and weighted.

### Calculation of narrow-sense heritability

Narrow-sense heritability was calculated using kinship matrix K, based on reconstructed genomes (i.e., probability of descent at about 11,000 genomic intervals). Narrow-sense heritability was estimated using a mixed model applied to the phenotype and kinship matrix *K* in which the phenotypic variance-covariance matrix V is modeled as $V=K{\sigma}_g^2+I{\sigma}_e^2$, where *I* is the identity matrix and ${\sigma}_g^2,{\sigma}_e^2$ is the genetic and environmental variance components that are estimated by the mixed model by maximum likelihood. The narrow-sense heritability is ${h}^2={\sigma}_g^2/\left({\sigma}_g^2+{\sigma}_e^2\right).$ Narrow-sense heritability estimates were calculated before and after correction for age, batch, and origin effects and quantile normalization.

### Calculation of broad-sense heritability

Broad-sense heritability (H^2^) including epistatic but not dominant effects was estimated using the proportion of phenotypic variance explained by differences in CC lines in a one-way ANOVA [39]. Broad-sense heritability was estimated by *Η*^2^ = *V*_*g*_/(*V*_*g*_ + *V*_*e*_), in which the environmental component of variance within lines is *V*_*e*_ = MS_within_ and the genetic component of variance among CC lines is *V*_*g*_ = (MS_between_ − MS_within_)/*n*, where *n* represents the average number of animals per line.

### Quantitative trait loci (QTL) analysis

Quantitative trait loci (QTL) mapping was performed using the HAPPY package [36]. Phenotype data were first corrected for age, batch, and origin effects and then quantile normalized, and CC line means were weighted by the number of animals per line. Analysis was performed at 11,000 genomic intervals, and significance thresholds were estimated by 1000 permutations. QTLs were considered significant at genome-wide permuted *P* < 0.05, and intervals were defined using a cutoff score of max Log*P*-1.

### Characterization of QTLs by homologous genes in humans

Human orthologous genes within each QTL interval were identified using BioMart build NCBIM37. Per QTL, we first compared the human orthologous genes with those reported in the SFARI human gene module (Additional file 1: Table S2). Secondly, although many ASD risk genes have been identified over the past decade and are included in the SFARI database, it seems likely that the SFARI genes still represent only a fraction of the total amount of ASD risk genes [40, 41]. Therefore, we also characterized genes within each QTL by their rank in a recently established ranking of all human genes by ASD implication by Krishnan et al. [40], adjusting for human genes without mouse ortholog. This approach is based on a validated machine-learning approach that combines prior genetic evidence with functional molecular interaction networks. Thirdly, we performed OMIM searches for each gene per QTL to identify genes associated with corresponding human phenotypes.

### Statistical analyses

All statistical analyses were performed with the statistical software R (R [42]).

## Results

### Structure of the genomes of CC lines in this study

We analyzed quantitative behavioral and physical variation in 53 CC lines available at Tel Aviv University (TAU), Israel (Fig. 1a) [27]. We first reconstructed the genome of each CC line as a mosaic of the eight CC founder strains, by calculating the probabilities of descent at about 11,000 genomic intervals using available genome-wide SNP array data from the MUGA and MegaMUGA platforms (Fig. 1b) [27]. In this set of CC lines, the overall genetic contribution of CC founder strains was within the expected range of about 1/8 = 12.5%, except for CAST/EiJ and PWK/PhJ, contributing 8.4 and 5.9%, respectively (Fig. 1c). Despite the lower overall abundance of these founder strains, complete absence of founder haplotypes was only observed at three genomic intervals, on chromosomes 2 and 8 (Fig. 1d). These results indicate that this set of CC lines represents a substantial mixture of the genetic diversity present in the eight CC founder strains.

### Quantitative variation in basic behavioral and physical traits

**Fig. 2:**
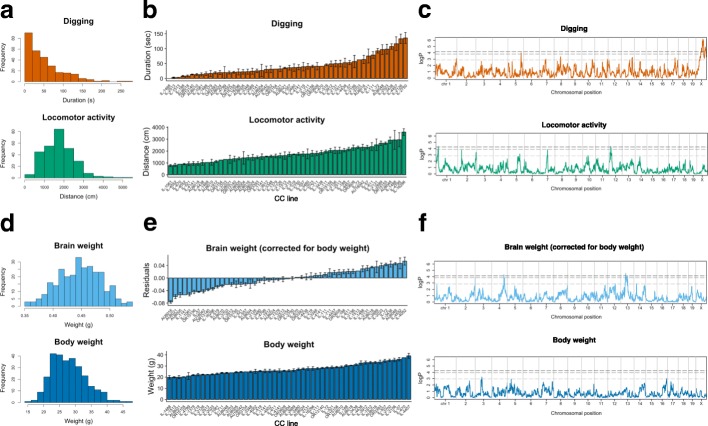
Quantitative variation in basic behavioral and physical phenotypes across CC lines and genetic mapping. **a** Histograms of digging and locomotor activity in 285 CC mice from 53 CC lines. **b** Digging and locomotor activity obtained from CC lines. Plots are expressed as mean ± SEM, with CC lines ordered along the *x*-axis by mean per phenotype (one-way ANOVA *P* = 2.1 × 10^−25^ and *P* = 1.1 × 10^−22^; one-way ANOVA after correction for age, batch, and origin *P* = 1.3 × 10^−17^ and *P* = 3.3 × 10^−15^). **c** Genome scans for digging and locomotor activity. Significance thresholds were estimated by 1000 permutations. QTLs were detected for digging on the X-chromosome (permuted *P* < 0.001) and for locomotor activity on chromosomes 1 and 12 (permuted *P* = 0.042 and *P* = 0.042). Dashed lines represent genome-wide permuted *P* < .05, 0.1, and 0.5. **d** Histograms of brain weight and body weight. **e** Brain weight (residuals after correction for body weight) and body weight obtained from 53 CC lines. Plots are expressed as mean ± SEM, with CC lines ordered along the *x*-axis by mean per phenotype (one-way ANOVA *P* = 1.1 × 10^−44^, *P* = 2.0 × 10^−48^; one-way ANOVA after correction for age, batch, and origin *P* = 1.1 × 10^−32^, *P* = 6.0 × 10^−30^). **f** Genome scans for brain weight (residuals after correction for body weight) and body weight. QTLs were detected for corrected brain weight on chromosomes 4 and 13 (permuted *P* = 0.034 and *P* = 0.021), but not for body weight

We first tested the influence of the genetic variation in these 53 CC lines on basic mouse behavioral traits and quantified digging and locomotor activity. The total population consisted of 285 male CC mice with 5–6 mice on average per CC line. We observed substantial continuous variation for these traits across the tested population (Fig. 2a), with significant differences between CC lines (Fig. 2b). We then estimated heritability and found that estimates of narrow-sense heritability (estimated from genome-wide genetic differences between lines) were substantial for digging and locomotor activity, both before (Fig. 4a, top) and after correction for age, batch, and origin effects and quantile normalization (Fig. 4b, top). Similar observations were made for broad-sense heritability (estimated from differences between CC lines) (Additional file 1: Figure S1c and d). To identify genetic loci contributing to the phenotypic variation observed after correction for age, batch, and origin effects and quantile normalization, we performed quantitative trait locus (QTL) analysis and identified significant QTLs for digging behavior on the X-chromosome and locomotor activity on chromosomes 1 and 12 (Fig. 2c and Additional file 1: Table S1).

To compare these basic mouse behavioral traits with general physical traits, we quantified brain weight and body weight and observed strong variation across CC lines (Figs. 2d, e). Pairwise Spearman’s rank-correlation analysis indicated a moderate correlation between brain weight and body weight (Additional file 1: Figure S2). Therefore, we calculated heritability estimates for total body weight and corrected brain weight, and observed consistently high heritability for these measures relative to basic mouse behavioral traits (Fig. 4a, b; top). We then performed QTL analysis (following correction for age, batch, and origin effects and quantile normalization) and identified loci associated with corrected brain weight on chromosomes 4 and 13. Perhaps surprisingly, we did not detect any locus for total body weight, despite high estimates of narrow-sense heritability (Fig. 2f). This is likely because many loci, each contributing a relatively small fraction of variance, contribute to body weight.

### Quantitative variation in phenotypes commonly used in animal models for ASD

**Fig. 3:**
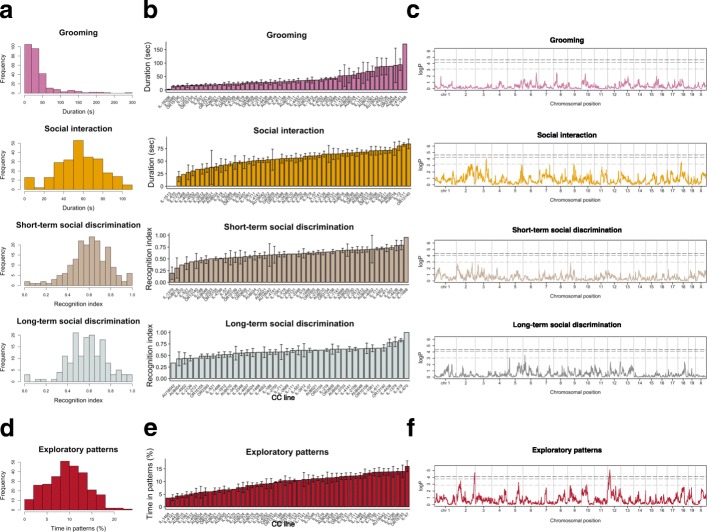
Quantitative variation in ASD-like phenotypes and genetic mapping in CC mice. **a** Histograms of grooming, social interaction, and short-term and long-term social discrimination. **b** Grooming, social interaction, and short-term and long-term social discrimination from up to 53 CC lines. Plots are expressed as mean ± SEM, with CC lines ordered along the *x*-axis by mean (one-way ANOVA *P* = 2.8 × 10^−5^, *P* = 2.8 × 10^−8^, *P* = 0.0035, and *P* = 0.33; one-way ANOVA after correction for age, batch, and origin *P* = 0.0027, *P* = 2.6 × 10^−5^, *P* = 0.18, and *P* = 0.31). **c** Genome scans for grooming, social interaction, and short-term and long-term social discrimination. No QTLs were detected. **d** Histograms of stereotyped exploratory patterns. **e** Stereotyped exploratory patterns obtained from CC lines. Plots are expressed as mean ± SEM, with CC lines ordered along the *x*-axis by mean per phenotype (one-way ANOVA *P* = 1.6 × 10^−14^; one-way ANOVA after correction for age, batch, and origin *P* = 3.3 × 10^−7^). **f** Genome scans for stereotyped exploratory patterns. QTLs for stereotyped exploratory patterns were detected on chromosome 2 (permuted *P* = 0.012) and chromosome 12 (permuted *P* = 0.003). Dashed lines represent genome-wide permuted *P* < .05, 0.1, and 0.5

**Fig. 4:**
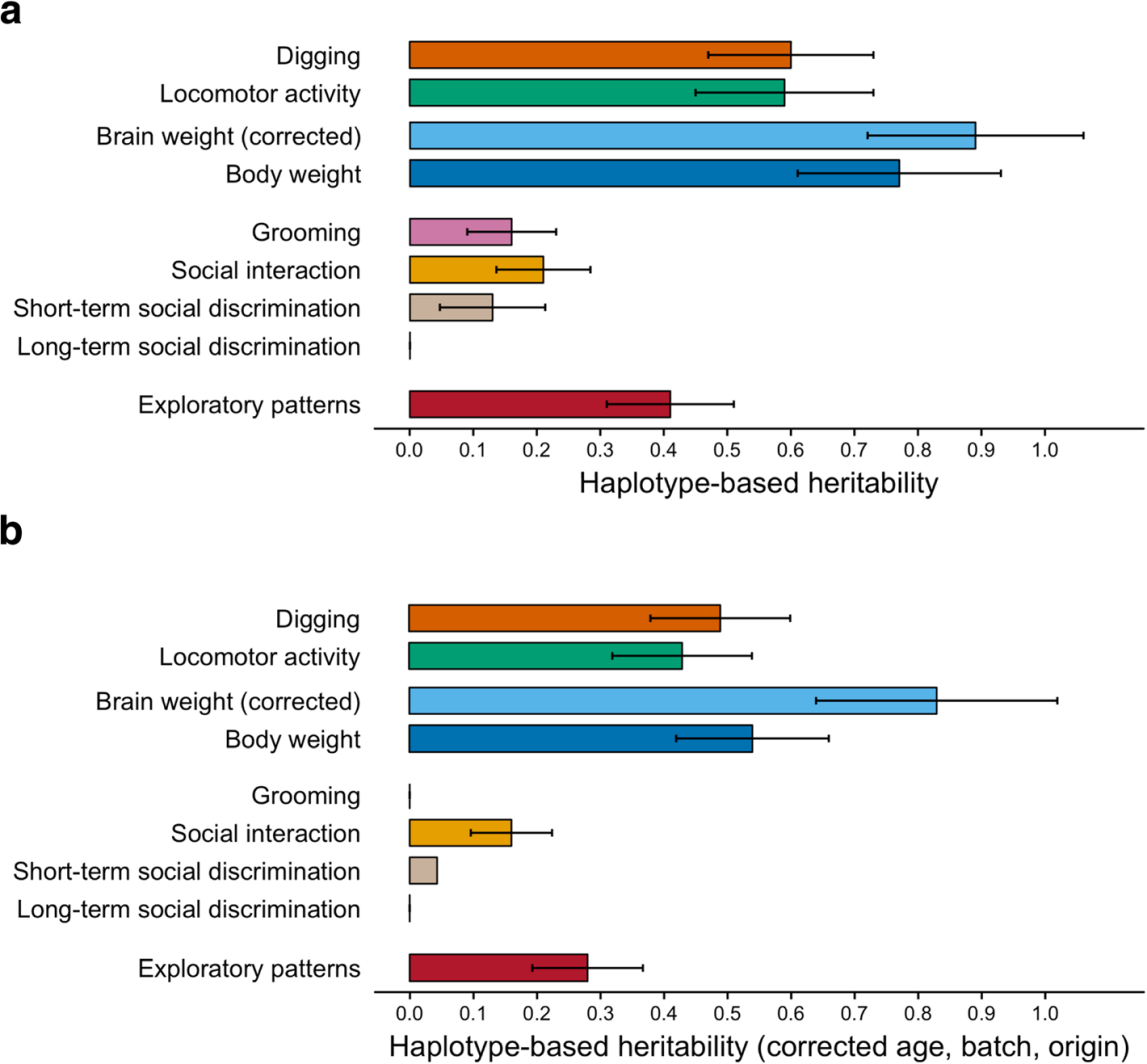
Haplotype-based heritability estimates in CC mice. **a** Genome-wide haplotype-based (narrow-sense) heritability estimates for basic mouse behavioral and physical traits (top), social behavior and grooming-related readouts (middle), and stereotyped exploratory patterns (bottom). **b** Genome-wide haplotype-based (narrow-sense) heritability estimates after correction for effects of age, batch, and origin and quantile normalization

Next, we analyzed behavioral traits commonly studied to establish face validity with human ASD and found continuous variation in social interaction, short-term, and long-term social discrimination (Fig. 3a) across CC lines. In contrast, for grooming behavior, we observed a more discrete distribution, indicated by a small fraction (6.0%) of animals with excessive levels (i.e., duration > 120 s) that were phenotypically separate from the majority (Fig. 3b). For all these ASD-related variables, differences within CC lines were relatively large, with modest differences between lines (Fig. 3b). We estimated heritability, both before and after correction for age, batch, and origin effects and quantile normalization, and found that estimates of narrow-sense heritability of ASD-related variables were low relative to the basic behavioral and general physical traits (Fig. 4a, b; middle). In contrast to the highly heritable phenotypes, no significant loci were identified in the QTL analysis for these low heritable phenotypes (social cognitive and grooming behavior variables) (Fig. 3c).

### Stereotyped exploratory patterns as a quantitative ASD-like phenotype

In search for mouse behavioral traits related to ASD with stronger genetic influences, we reverted to the BTBR mouse inbred strain to find candidate phenotypes that may yield more genetic contrast in the CC lines. Following the variation in digging and grooming behavior in our CC population, we hypothesized that ASD phenotypes relating to lower order repetitive behavior might show stronger genetic control. The BTBR mouse inbred strain has a wide variety of natural genetic variation compared to other mouse laboratory inbred strains (e.g., C57BL/6 J) and is commonly used as a mouse model for ASD with predominant abnormalities in behavioral inflexibility and repetitiveness [16–18, 43]. We observed that BTBR mice also display high amounts of stereotyped exploratory patterns and quantified the total time in these stereotyped exploratory patterns (Additional file 1: Figure S3a) [38]. Using this quantification of stereotyped and repetitive behavior, we confirmed a strong difference in the total time spent in patterns between BTBR and C57BL/6J mice (Additional file 1: Figure S3b), which remained highly significant after correcting for strain differences in locomotor activity levels (Additional file 1: Figure S3c).

Following the characterization of stereotyped exploratory patterns during novelty exposure in BTBR mice, we tested this phenotype in our population of CC mice. We found that this readout was continuously distributed with substantial variation (Fig. 3d) and was characterized by substantial differences between CC lines (Fig. 3e). Moreover, estimates of broad- and narrow-sense heritability for stereotyped exploratory patterns were intermediate when compared to relatively high heritability phenotypes (e.g., body weight) and to relatively low heritability phenotypes (e.g., social discrimination) (Fig. 4a, b; bottom).

### Stereotyped exploratory patterns and Hrh3

Next, we performed QTL analysis of stereotyped exploratory patterns, which led to the identification of significant QTLs on chromosomes 2 and 12 (Additional file 1: Table S1), and QTLs with suggestive evidence on chromosomes 2, 5, and 10 (Fig. 2f). Interestingly, the QTL for stereotyped exploratory patterns on chromosome 2 contained *Hrh3* (Additional file 1: Table S2), a histamine signaling gene implicated in ASD by expression-analysis in post-mortem brains [44]. Moreover, activation of the histamine receptor H3R in the dorsal striatum was recently shown to trigger motor stereotypies in mice, and antagonism of H3R was found to attenuate elevated repetitive behavior in an animal model of autism induced by prenatal exposure to valproic acid [45, 46].

### Intersection of genes in QTLs with human neurodevelopmental risk genes

As further proof of principle, we also investigated whether QTLs for basic behavior and brain phenotypes (Additional file 1: Tables S1–2) were enriched for genes relevant to NDD- and ASD-like phenotypes in humans. Although many risk genes have been identified for ASD over the past decade, it seems likely that these genes still only represent a fraction of the total amount of ASD risk genes [40, 41]. For that purpose, we identified the human orthologs of genes within each mouse behavioral QTL and ranked these genes on the basis of a recently developed genome-wide prediction algorithm to prioritize genes for ASD based on prior genetic evidence and functional interaction networks [40]. Following this strategy, we found that homologous genes in the QTL for locomotor activity on chromosome 1 showed enrichment of genes with high ASD ranking (Additional file 1: Figure S4, *P* = 0.02). High ASD-ranked genes within this QTL included brain-specific angiogenesis inhibitor 3 (*Bai3*), a member of the BAI subfamily of adhesion G-protein-coupled receptors (GPCRs). These GPCRs regulate synapse development and plasticity and are implicated in various neurological and psychiatric disorders [47–49].

Moreover, the QTL for brain weight on chromosome 4 contained two genes, one of which codes for HMGB4, which is known to be highly expressed in neural stem cells and serves as a regulator of chromatin and neural differentiation markers [50, 51]. Furthermore, the QTL for brain weight on chromosome 13 contained *Atxn1*, a gene involved in neural proliferation [52], whose disruption is known to reduce cortical thickness in mice and to cause a spectrum of neurobehavioral phenotypes including hyperactivity in both mice and humans [53]. The overlapping region of loci for exploratory patterns and locomotor activity on chromosome 12 included a mouse homolog of the human *YWHAE* gene, implicated in the Miller-Dieker syndrome that is located on chromosomal position 17p13.3 [54, 55] (also see [56]). Finally, although the QTL for digging behavior on the X-chromosome was very broad, notable genes in this locus included *Dc*x, coding for neuronal migration protein doublecortin, and *Htr2C* gene, coding for a serotonin receptor implicated in mental illnesses including OCD and depression [57–60].

## Discussion

These results showed that CC recombinant inbred lines can complement existing genetic or inbred animal models for neurodevelopmental disorders. The current sample of CC inbred lines showed a population distribution of phenotypic variability for multiple NDD traits, and we identified QTLs that harbor genes strongly associated with corresponding NDD phenotypes in humans. Overall, the results indicated that CC lines can be used to study the quantitative impact of genetic manipulations and environmental perturbations on the basis of controlled genetic background variability.

The obtained results in this CC population highlight that the choice of behavioral phenotypes is crucial to mimic trait dynamics and genetic associations observed in population studies [3]. First, phenotypes should be continuously distributed traits in the study population. We found that grooming behavior was non-continuously distributed in the CC population, indicating that this phenotype may only capture genetic effects in a smaller proportion of the population. Secondly, phenotype expression should be heritable in the study population, similar to ASD- and ADHD-like traits in the general human population. In this respect, locomotor activity, a measure of stereotyped exploratory patterns and social interaction seemed more suitable compared to low heritability traits such as short-term and long-term social discrimination. As observed from human genetic population studies, larger sample sizes will be required to identify genetic loci for highly “diluted” phenotypes (e.g., body weight and social interaction) and to be able to detect loci with very small effect size.

Once these criteria have been met, translational value may be indicated by the intersection of human neurodevelopmental risk genes with genetic associations of homologous genes in mouse populations. The current set CC lines appeared insufficient to resolve genetic loci at a single-gene level. We were, however, able to identify multiple QTLs and show that several genes in the identified QTLs are implicated in normal and abnormal brain development and are enriched for genes that are associated with ASD.

In this study, we identified two QTLs for locomotor activity on chromosomes 1 (23.484–29.342 Mb) and 12 (16.824–26.491 Mb). We found that the locus on chromosome 1 was enriched for high ASD-ranking genes and that the highest ASD-ranking gene in this QTL was *Bai3*. This gene has been associated with predisposition for schizophrenia and addiction and is considered a potential target for pharmacological intervention [47–49, 61]. While previous studies have identified QTLs for locomotor activity also on mouse chromosomes 1 and 12, we did not observe any overlap between our QTLs in CC mice and the QTLs for locomotor activity that have been reported in previous studies in different samples of mice (for a review see [13]). For example, multiple loci for activity in a new home-cage were identified in heterogeneous stock mice by [19], including multiple QTLs on chromosome 1 (3.539–6.621 Mb and 105.835–109.516 Mb (build 34)) and on chromosome 12 (59.029–62.702 Mb and 95.300–95.652 Mb (build 34)), that do not overlap with our QTLs in CC mice. In addition, our QTLs for locomotor activity do not overlap with genetic loci for open field activity derived from F2 intercrosses of A/J and C57BL/6J mice [62], of DBA/2 and C57BL6/J mice [63], and of DeFries strains of mice [64]. We also find no overlap between our QTLs for locomotor activity with those that have been reported in BXD recombinant inbred lines, such as a suggestive QTL for open field activity on chromosome 4 [21] and a locus derived from locomotor activity during the habituation phase of a social interaction paradigm on chromosome 12 (88.695–99.431 Mb) [22]. The variety of non-overlapping QTLs in different studies for similar behavioral traits (i.e., measures of locomotor activity) could be the result of evident differences in behavioral testing procedures and protocols. Moreover, another possible explanation for this observation could be the fact that CC lines are derived from a wide range of founders, including three wild-derived strains.

We identified two QTLs for brain weight unrelated to body weight on chromosomes 4 and 13. Both loci contained genes known to be involved in brain development, including *Atxn1* that plays a critical role in neural proliferation and cortical development [52, 53], as well as *Hmgb4*, which regulates the expression of neuronal differentiation markers.

The X-chromosome was highly associated with digging behavior in CC mice, which was indicated by three QTLs for this behavior on this chromosome. While these loci contained many genes, this association is an interesting observation because many genetic neurodevelopmental disorders are X-linked. Moreover, in mice, the X-chromosome has been implicated in autistic-like symptoms in a study that derived behavioral QTLs from intercrossing C57BL/6J and the BTBR mice [15]. We observed that one of our three QTLs for digging behavior in CC mice (105.265–126.750 Mb) maps onto a broad QTL for “novel mouse sniffing” derived from BTBR mice (5.996–132.120 Mb). However, the overlapping region did not contain the *Htr2C* gene, which is known to cause repetitive behavior symptoms in a gene knockout model [57, 65]. We did not observe any other overlap between our neurobehavioral QTLs in CC mice, and QTLs for autistic-like behaviors derived from BTBR mice [15]. Moreover, we did not observe overlap between our QTLs and those reported by [22] for social interaction and ultrasonic vocalizations derived from BXD mice.

We identified a gene coding for H3 receptor in a QTL for repetitive behavior patterns. Manipulation of this receptor has been shown to alter repetitive behavior expression both in an anatomical model as in a pharmacological model [44–46]. For example, the H3 receptor is highly expressed in striatal nuclei, and histamine H3 receptor activation in the dorsal striatum was recently shown to trigger stereotypies in mice [46]. Recently, a novel potent and selective H3 receptor antagonist was found to attenuate stereotyped repetitive behaviors in genetically diverse sample of mice that were prenatally exposed to valproic acid [66].

Taken together, our findings highlight the potential to use the CC population to assess the quantitative impact of genetic manipulations and pharmacological compounds on behavioral dimensions across different genetic backgrounds, e.g., as a genetic reference population. The use of CC for NDD studies can be expanded into more targeted experiments, for example, by heterozygous crosses between CC lines and gene knockout lines [30], or by CRISPR/Cas9-mediated genome editing in a subset of CC lines to quantify the impact of genetic mutations observed in human populations across genetic backgrounds [67].

## Conclusions

Quantitative, population-based approaches in animal modeling are needed to complement models based on single genetic mutations in a single background. Our results provide a starting point for modeling the quantitative nature of NDD using a genetic reference population and demonstrate a unique potential of CC lines for future studies into mouse models of neurodevelopmental disorders.

## Additional file

Additional file 1: **Figure S1.** Haplotype-based and broad-sense heritability estimates in CC mice. **Figure S2.** Phenotypic correlations in CC mice. **Figure S3.** Quantification of stereotyped exploratory patterns in C57BL/6J and BTBR mice. **Figure S4.** Characterization of genes in QTL by predicted ASD implication. **Table S1.** Quantitative trait loci (QTLs) for neurobehavioral traits in CC mice. **Table S2.** Human homologous genes in QTLs derived from CC mice. (DOCX 8519 kb)

## Abbreviations

ADHD: Attention-deficit/hyperactivity disorder
ASD: Autism spectrum disorders
CC: Collaborative Cross
NDD: Neurodevelopmental disorders
QTLs: Quantitative Trait Loci
RI lines: Recombinant Inbred lines
