# Supplementary material for: Modeling the quantitative nature of neurodevelopmental disorders using Collaborative Cross mice
Source: Mol Autism. 2018 Dec 13;9:63. doi: 10.1186/s13229-018-0252-2 (PMC6293525; doi:10.1186/s13229-018-0252-2)
Supplement: Supplementary file 1 — Figure S1. Haplotype-based and broad-sense heritability estimates in CC mice. Figure S2. Phenotypic correlations in CC mice. Figure S3. Quantification of stereotyped exploratory patterns in C57BL/6J and BTBR mice. Figure S4. Characterization of genes in QTL by predicted ASD implication. Table S1. Quantitative trait loci (QTLs) for neurobehavioral traits in CC mice. Table S2. Human homologous genes in QTLs derived from CC mice. (DOCX 8519 kb) [file 13229_2018_252_MOESM1_ESM.docx]

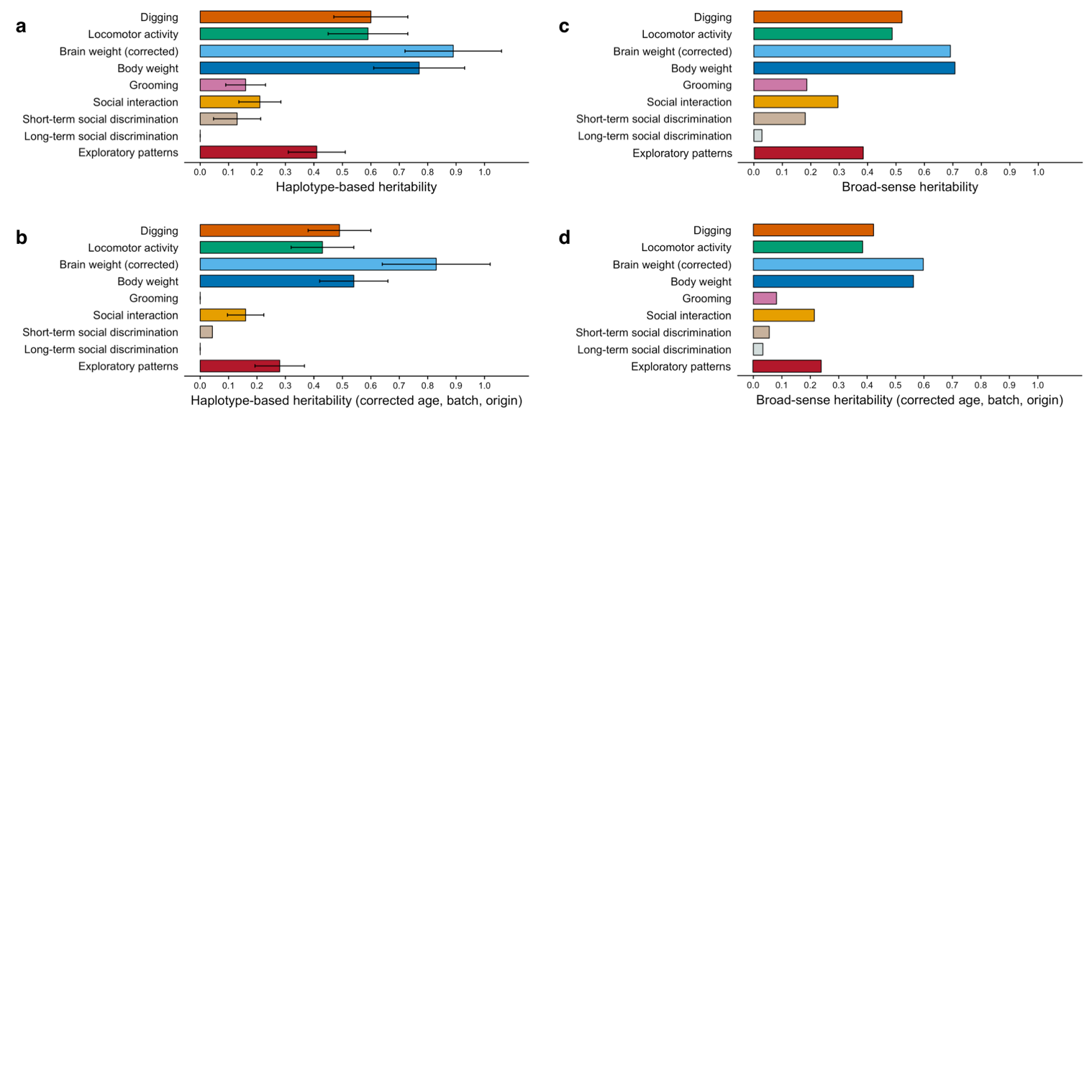


***Figure S1. Haplotype-based and broad-sense heritability estimates in CC mice.***

***(a)*** *Genome-wide haplotype-based heritability estimates for basic mouse behavioral and physical traits (top), social behavior and grooming-related readouts (middle), and stereotyped exploratory patterns (bottom).*

***(b)*** *Genome-wide haplotype-based heritability estimates after correction for effects of age, batch and origin, and quantile normalization.*

***(c)*** *Broad-sense heritability estimates for basic mouse behavioral traits (top), physical traits (middle), and ASD-related readouts (bottom).*

***(d)*** *Broad-sense heritability estimates after correction for effects of age, batch and origin, and quantile normalization.*

**

***Figure S2. Phenotypic correlations in CC mice.***

*Spearman’s rank correlation coefficients for each pair of variables (calculated using all complete pairs of observations on those variables). Blue colors indicate positive correlations, and red colors indicate negative correlation.*

*
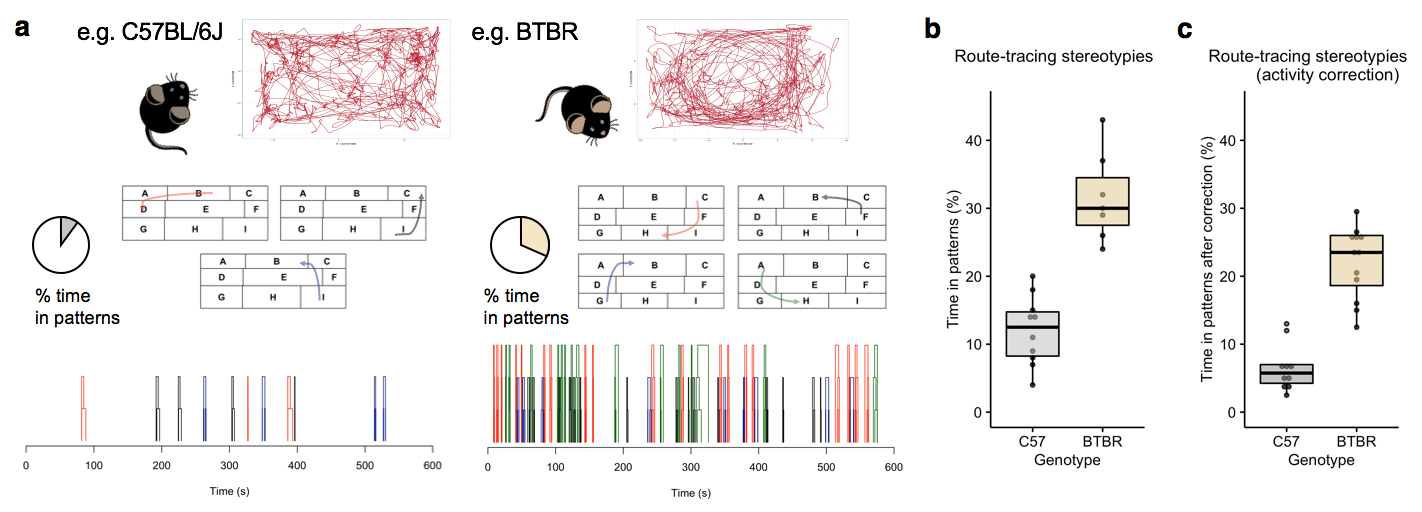
*

***Figure S3. Quantification of stereotyped exploratory patterns in C57BL/6J and BTBR mice.***

***(a)*** *Example of exploratory patterns during novelty exposure in C57BL/6J mice and the BTBR inbred strain models for ASD. Exploratory patterns were quantified following Bonasera et al. 2008, by testing whether spatial bins of locomotor activity occur in particular sequences (‘patterns’) over time at a probability greater than chance. Following the identification of patterns (e.g. red, green, black, blue) with different levels of complexity, scores for exploratory patterns are defined as the total percentage of time the animal spent in patterns (e.g. 7% in C57BL/6J vs. 22% in BTBR).*

***(b)*** *Box-plot comparison of exploratory patterns in C57BL/6J and BTBR mice (Welch’s t-test t(10.83) = 6.63, P=4.0x10^-5^).*

***(c)*** *Exploratory patterns after activity-correction (C57BL/6J (n=10) and BTBR mice (n=12), Welch’s t-test t(19.07) = 8.20, P=1.1x10^-7^). Activity correction was applied by considering equal distances across all animals, i.e. first and last 1500cm in C57BL/6J and BTBR mice.*


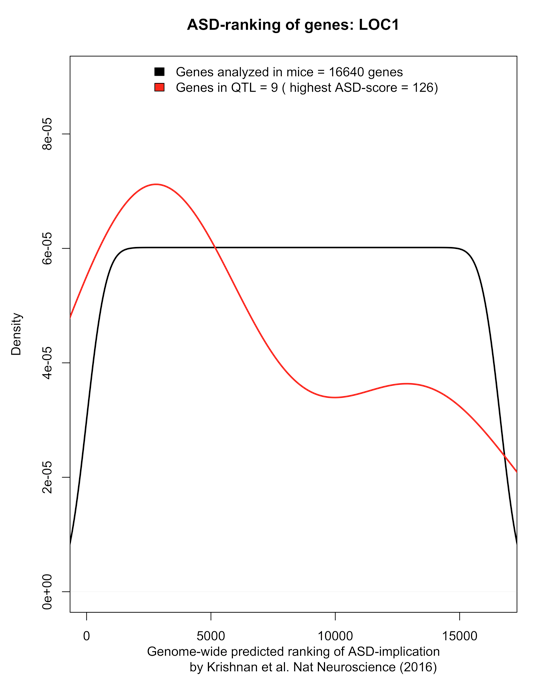


***Figure S4. Characterization of genes in QTL by predicted ASD-implication.***

*Proof of principle comparison of the 9 genes in the locomotor activity QTL on chromosome 1, to predicted ranking of all human genes by ASD-implication (Krishnan et al. Nat Neurosci 2016). Genes in this QTL show higher density for high-ranking genes compared to all genes with mouse-homolog (highest ASD-ranking gene ranked 124^th^, median 3476^th^). Equal or smaller median values were observed in fraction 0.021 of 10000 equally-sized randomly sampled gene sets.*

***Table S1. Quantitative trait loci (QTLs) for neurobehavioral traits in CC mice.***

| **Phenotype** | **QTL** | **Location**  **(Mb, build 37)** | **Max logP** | **Permuted P** | **Width (Mb)** | **Homologous genes** |
| --- | --- | --- | --- | --- | --- | --- |
| Digging | DIG1 | X:105.265-126.750 | 6.19 | P<0.001 | 21.5 | 26 |
|  | DIG2 | X:163.579-165.659 | 6.14 | P<0.001 | 2.1 | 7 |
|  | DIG3 | X:140.042-158.193 | 4.89 | 0.012 | 18.2 | 63 |
| Locomotor activity | LOC1 | 1:23.484-29.342 | 4.33 | 0.042 | 5.9 | 9 |
|  | LOC2 | 12:16.824-26.491 | 4.35 | 0.042 | 9.7 | 27 |
| Brain weight | BRW1 | 4:127.229-128.178 | 4.33 | 0.034 | 0.9 | 2 |
|  | BRW2 | 13:45.602-55.115 | 4.54 | 0.021 | 9.5 | 69 |
| Exploratory patterns | EXPL1 | 2:173.252-179.848 | 4.69 | 0.012 | 6.6 | 27 |
|  | EXPL2 | 12:17.282-28.665 | 5.07 | 0.003 | 11.4 | 25 |

***Table S2. Human homologous genes in QTLs derived from CC mice.***

| **QTL** | **Human homologous genes in QTL** |
| --- | --- |
| DIG1 | *APOOL, BRWD3, CHM, CPXCR1,* ***CYLC1****, DACH2, DMTF1, DPPA5, HDX, HMGN5,* ***KLHL4****, MIR361,* ***NAP1L3****, PABPC5,* ***PCDH11X*****, PCDH11Y, POF1B, POU3F4,* ***RPS6KA6****, SATL1, SCARNA4, SH3BGRL, SNORA9, TGIF2LX, TGIF2LY, ZNF711* |
| DIG2 | *AMELX, AMELY, FRMPD4*, PRPS2, TLR7, TLR8, TMSB4Y* |
| DIG3 | *ACOT9, ALAS2,* ***AMOT****, APEX2, CAPN6, CDKL5*,* ***CNKSR2*****, CXorf23****, DCX*****, FAM120C, FAM156A, FAM156B,* ***FGD1*****, FOXR2, GNL3L, GPR143, GPR173, GPR64, HSD17B10,* ***HTR2C****, HUWE1*, IL13RA2, IQSEC2*, KDM5C*, KLF8, KLHL34, LHFPL1,* ***LRCH2****, MAGED2, MAGEH1, MAP3K15, MAP7D2, MBTPS2, MIR98, PDHA1, PFKFB1, PHEX, PHF8*, PHKA2, PPEF1, PRDX4, PTCHD1*, RIBC1****, RPS6KA3*****, RRAGB,* ***RS1****, SAT1, SCML2, SH3KBP1*,* ***SHROOM2****, SMC1A*, SMPX, SMS, SNORA32, SNORA35, SNORA4,* ***TRO****, TRPC5,* ***TSPYL2****, TSR2,* ***UBQLN2****, USP51, ZCCHC16* |
| LOC1 | *B3GAT2,* ***BAI3,*** *C6orf57, COL19A1,* ***COL9A1,*** *FAM135A, LMBRD1, SMAP1, SNORA64* |
| LOC2 | *ADAM17, ASAP2, ATP6V1C2, C2orf50, CPSF3, CYS1, GRHL1,* ***HPCAL1****, IAH1, ID2, ITGB1BP1,* ***KCNF1****,* ***KIDINS220****, KLF11,* ***MBOAT2****, NOL10, ODC1, PDIA6, PQLC3, ROCK2, RRM2, SNORA21, SNORA32, SNORA70, TAF1B,* ***YWHAQ****, ZNF124* |
| BRW1 | *C1orf94, HMGB4* |
| BRW2 | *ARL10, ASPN,* ***ATXN1****, AUH, BARX1, BICD2, C9orf89,* ***CAP2****, CDHR2, CENPP, CKS2,* ***CLTB****,* ***CPLX2****,* ***DCUN1D1*****,* ***DEK****,* ***DIRAS2****,* ***DRD1*****, ECM2, EIF4E1B, FAF2, FAM120A, FAM8A1, FGD3, GADD45G, GPRIN1, HIGD2A, HIGD2B, HRH2, IARS,* ***ID4****, IPPK, KDM1B, KIAA1191, KIF13A,* ***MSX2****, NFIL3, NHLRC1, NINJ1, NOL8, NOP16,* ***NUP153****, NXNL2, OGN, OMD,* ***PHF2*****, PTPDC1, RBM24,* ***RNF144B****, RNF44***,** *ROR2, S1PR3, SECISBP2, SEMA4D, SFXN1,* ***SHC3****,* ***SNCB****, SNORA24, SNORA55, SNORA84, SPIN1, SPTLC1, SUSD3, SYK, THOC3, TPMT, TSPAN17*, UNC5A, WNK2, ZNF169* |
| EXPL1 | *ANKRD60, ATP5E, ATP5EP2, C20orf85, CDH26,* ***CDH4****, CTSZ,* ***EDN3****, FAM217B, GNAS*,* ***HRH3****, LSM14B, MIR298, NPEPL1, PHACTR3, PPP1R3D,* ***PSMA7****, RAB22A, SLMO2,* ***SS18L1****,* ***STX16****, SYCP2,* ***TAF4****, TUBB1, VAPB, ZNF124, ZNF831* |
| EXPL2 | ADAM17, ASAP2, ATP6V1C2, CMPK2, CPSF3, CYS1, GRHL1, **HPCAL1**, IAH1, **ID2**, ITGB1BP1, **KIDINS220**, KLF11, MBOAT2, NOL10, ODC1, RNF144A, RRM2, RSAD2, SNORA21, SNORA70, **SOX11**, TAF1B, **YWHAQ**, ZNF124 |

** represents genes scored in the SFARI database;* ***bold*** *represents genes found in the top decile of predicted ASD-ranking by Krishnan et al. 2016. Genes are listed in alphabetical order.*
